# Supplementary material for: Are carbon emissions trading and green financial instruments synergistic? -Comprehensive quantitative research based on content analysis
Source: PLoS One. 2024 Mar 7;19(3):e0298601. doi: 10.1371/journal.pone.0298601 (PMC10919601; doi:10.1371/journal.pone.0298601)
Supplement: S1 Data — (ZIP) [file pone.0298601.s001.zip › Data/地级市Z_F工作报告.zip!/╔╜╢1⁄2/║╩╘≤╩╨/└┤╫╘╣1⁄2╓┌║┼í╛┬φ┐╦╩2╛▌═°í┐_2007.docx]

各位代表:

　　现在,我代表市人民政府向大会作政府工作报告,请予审议,并请市政协委员和其他列席人员提出意见。

　　一、2006年工作回顾

　　过去的一年,在中共菏泽市委的坚强领导下,全市人民认真贯彻科学发展观和国家宏观调控政策,全面落实市委“一二三四”的工作思路,解放思想,干事创业,圆满完成了市十六届人大四次会议确定的各项任务。

　　国民经济快速健康发展。实现地区生产总值537.7亿元,比上年增长17.1%,高于全省2.4个百分点,高于“十五”时期平均增幅4个百分点。完成地方财政收入30亿元,可比增长47.4%,工商税收占地方财政收入比重由上年的69%提高到72%。全部税收收入完成43.2亿元,增长43%,其中国税23.7亿元、地税19.5亿元,分别增长34%和54%。各项存款余额496.5亿元,各项贷款余额397.5亿元,分别比上年初增加70.5亿元和58.5亿元,均创历史新高。万元生产总值能耗、化学需氧量排放量分别下降4.5%和5%,城区空气质量良好率达到87.4%。

　　经济结构进一步优化。三次产业比调整为31:46:23,二三产业比重提高3.3个百分点。工业化进程明显加快,规模以上工业企业达到1043家,增加222家;完成增加值170.5亿元,增长29.4%;实现利税42.1亿元、利润21.5亿元,分别增长49.9%和53.5%。服务业发展迅速,实现社会消费品零售总额275.3亿元,增长16.3%。完成进出口总额6.5亿美元,增长31.2%。50个服务业重点项目规划、建设进展顺利。

　　新农村建设全面推进。粮食总产47.9亿公斤,增长16.8%。森林覆盖率提高到28.9%,肉、蛋产量分别增长6.1%和1.8%,农业机械化综合水平达到68%。新增规模以上农产品加工企业50家、农民专业合作经济组织189个,农产品标准化基地达到150万亩,带动120万农户。国有粮食购销企业改革顺利完成。“四四四一”工程初见成效,改厕25.6万座,改灶2.1万户,新建沼气池8700个,新增农村油路1400公里,新增农村自来水受益人口83万人,培训农民15万人次,新农村建设试点乡镇、试点村的规划基本完成。

　　城市和基础设施建设取得重大进展。编制完成了市区中心区、老城区控制性详细规划、东部新区分区规划以及消防、绿地水系、路网、电力等专业规划,市区详规覆盖率提高10个百分点。大力推进城市道路、污水管网、绿化亮化、供水供热供气、安康居住和综合开发工程,完成政府投资4亿元,社会投资22亿元。环城公园获得中国人居环境范例奖。环卫改革继续深化,城市管理日益加强,市容市貌有了新的改观。着力加强基础设施建设,德商高速南段及黄河公路大桥开工建设,菏关高速即将通车,济菏高速及干线公路改造工程进度加快。鄄城220千伏输变电工程一次送电成功,洙水河航道改造工程启动建设,谢寨、闫潭送水干线清淤工程顺利完成。

　　固定资产投资水平不断提高。加大项目报批力度,利用东西帮扶机制,创新招商引资方式,密切与省金融机构战略合作,实现了固定资产投资健康增长。全市完成规模以上固定资产投资500.4亿元,增长22.1%,其中招商引资320亿元,占64%;外商直接投资7090万美元,增长86.9%。重点项目建设成效显著,巨野煤田5对矿井建设顺利推进,彭庄矿井投入试生产;菏泽电厂三期、单县生物发电、东明石化100万吨延迟焦化、艺达家纺一期、达驰节能变压器、百隆纺织二期、100万吨山水水泥、40万吨洋丰复合肥等重点项目相继竣工投产。一批亿元以上的重点项目正加紧施工。园区建设取得重大进展,累计完成投资280亿元。

　　各项社会事业加快发展。科技创新能力进一步增强,取得重要科技成果98项。完成投资1.1亿元,改造农村中小学危房24万平方米,4所市区小学改扩建工程基本竣工;适龄儿童小学入学率达到99.8%,小学毕业生升学率达到98.1%;本科录取人数1.1万人,增长10%。引进具有博士、硕士学位的高学历人才155人。《山东汉子》在30台国家舞台艺术精品工程评选中取得第12名的好成绩。积极推进农村广播电视村村通工程,10万户农民收看到了有线电视节目。公共卫生体系建设不断加强,四个新农合试点县农民参合率达到88.6%,受益人口300多万人。竞技体育实现新突破,在第21届省运会上,我市获得金牌总数比上届前移3个位次。人口自然增长率控制在4.2‰以内,圆满完成了年度人口责任目标。精神文明创建活动深入开展。军政军民团结更加巩固。新闻出版、社会科学、民族宗教、妇女儿童、外事侨务、档案管理、人防、气象、地震、史志、老龄、残疾人等事业都有新的发展。

　　和谐社会建设迈出新步伐。新增劳务输出20.3万人,新增城镇就业人口11万人,城镇登记失业率为3.5%;农民人均纯收入达到3480元,城镇居民人均可支配收入达到8137元,分别增长12.5%和10.8%。征缴各类保险费12亿元,确保了企业离退休人员养老金和失业人员失业金按时足额发放;妥善解决了8000名市属困难企业下岗职工遗留问题;全面提高了城镇低保、伤残军人、农村五保户等补贴标准,开展了对一些特殊困难群体的救助活动。发放“两免一补”资金1591万元,救助贫困生12.8万人。开工建设经济适用房1428套,其中619套已完成主体工程。公务员登记工作进展顺利。深入开展严打整治斗争,积极推进平安菏泽建设,重大刑事案件发案率继续下降。安全生产管理工作不断强化,圆满完成了各项省控指标。

　　政府自身建设进一步加强。扎实推进责任政府、诚信政府、阳光政府和法治政府建设,政府的执行力和公信力有所提高。推行重大事项公示、听证和专家咨询制度,政府决策机制更加完善。实行周安排月调度制度,促进了政府工作落实。深入开展争创一流活动,大项目争取、劳务输出、污水处理厂建设、农村改厕、经济适用房建设、环卫体制改革、敬老院建设等工作取得显著成效。自觉接受人大法律监督和政协民主监督,加强行政内部监督,主动接受社会、群众和新闻监督。严格落实党风廉政建设责任制,深入开展纠风专项治理活动,解决损害群众利益的突出问题取得新的成效。

　　各位代表!过去的一年,全市经济社会发展取得了丰硕成果,实现了“十一五”良好开局。这是省委、省政府实施“突破菏泽”战略的结果,是市委总揽全局、正确领导的结果,是人大、政协和社会各界有效监督、大力支持的结果,是全市人民团结拼搏、扎实奋斗的结果。在此,我代表市人民政府,向全市广大干部群众,向各民主党派、工商联、人民团体和各族各界人士,向驻菏部队、武警官兵和中央、省属单位,向所有关心支持菏泽发展的朋友们,表示崇高的敬意和衷心的感谢!

　　各位代表!过去一年取得的成绩令人鼓舞,但前进道路上还有不少矛盾和问题。主要是:经济总量小,工业化水平低,大项目、骨干企业和名牌产品少;企业创新能力差,经济增长方式粗放,节能减排任务艰巨;国家继续实施宏观调控,经济发展面临的土地紧张、资金短缺等问题更加突出;城镇化水平低,农村基础设施薄弱,农业产业化发展慢,农民增收难度大;财政困难,统筹发展能力弱;社会事业欠帐多,就业压力大,社会保障水平低;市、县政府组织经济社会发展的能力不够强,工作中存在诸多缺点、不足和薄弱环节,有的政府部门在工作作风、服务质量和行政效率等方面还存在不少问题。对此,我们一定认真对待,采取有力措施,切实加以解决。

　　二、2007年工作的总体要求和主要任务

　　2007年,是落实科学发展观、构建和谐社会的重要一年,也是实施“突破菏泽”战略、推动全市经济社会又好又快发展的关键一年。综合分析国内外形势,我们既面临着良好的发展机遇,也面临着严峻的挑战。根据省里的部署和市委关于站在新起点、谋求新发展、实现新突破的要求,今年政府工作的总体要求是:以邓小平理论和“三个代表”重要思想为指导,深入学习贯彻党的十六大及各次全会精神,全面落实科学发展观,坚持以人为本,积极构建和谐社会,坚持“一二三四”的工作思路,更加注重速度和结构、质量、效益、后劲相统一,更加注重节能降耗、自主创新、环境保护和土地集约利用,更加注重经济社会民生、改革发展稳定相协调,进一步加强作风建设,提高工作水平,全面实现“三三一一”目标,确保全市经济社会又好又快发展。全年经济社会发展的主要预期目标是:生产总值增长15%;规模以上工业增加值增长30%;地方财政收入增长20%;固定资产投资增长20%;社会消费品零售总额增长18%;外贸进出口增长18%;外商直接投资增长15%;万元生产总值能耗下降4.5%,规模以上工业万元增加值能耗下降4.7%;化学需氧量排放量降低5%;城镇居民人均可支配收入和农民人均纯收入分别增长9%和8%;城镇登记失业率控制在4%以内;居民消费价格总水平涨幅控制在3%以内;人口自然增长率控制在6.9‰以内。

　　围绕实现上述目标,重点抓好以下几项工作:

　　(一)着力提高投资质量和效益。积极搞好项目储备。根据国家产业政策,围绕培育六大产业、建设三大基地和发展服务业,策划论证一批大项目,重点策划论证3个2×60万千瓦电厂、东新高速、万福矿井、巨野60万吨甲醇、广源6万吨精密铜带、东明石化日东输油管道、开发区50万吨尿素、玉皇对二甲苯等26个投资过10亿元的项目,积极做好申报争取工作。力争年内审批、核准或备案的项目达到500亿元,进入上级计划盘子的项目数占全省10%以上。

　　全力推进大项目建设。加快建设三利纺织一期、东明石化15万吨离子膜烧碱、健民制药二期、达驰非晶合金变压器、单县化工1.5万吨橡胶促进剂、黄河纺织22万纱锭等40个投资过亿元的续建项目,争取早日竣工投产。加快巨野煤田矿井建设,确保彭庄煤矿一季度正式投运,龙土固、赵楼煤矿四季度试生产。早日开工建设开发区40万吨甲醇、洪业化工10万吨己内酰胺、艺达家纺二期、睿鹰制药2亿支针剂、圣奥化工RT培司扩建、巨野国能生物发电等50个投资过亿元的项目。

　　努力提高招商实效。按照“六个转变”的要求,健全招商网络,改进招商方式,突出抓好以商招商、网上招商、代理招商和产业招商。继续办好牡丹花会和中国林交会,精心组织各类招商活动,搞好项目对接,力争全年引进市外资金400亿元。做好企业境内外上市工作,争取年内有2家企业成功上市。

　　切实加强园区建设。主动争取东部强市支持,继续加大园区投入,增强载体功能。按照功能互补、产业关联、科学布局的原则,引导项目向10个工业园区集中,加速形成产业聚集效应,推动项目尽快投产达产。认真执行国家土地政策,严格落实投资强度等控制性指标,搞好存量建设用地挖潜,提高节约集约用地水平。园区完成固定资产投资300亿元,其中基础设施投资10亿元。积极争取将牡丹工业园列入省级高新区。

　　(二)加快推进工业化进程。大力培植骨干企业。全面加强企业家队伍建设,进一步提高企业家干大事、创大业的能力和水平,尽快培养出在全省乃至全国知名的骨干企业和优秀企业家。进一步完善政策措施,在财政税收、项目用地、融资担保、人才引进、基础设施建设等方面加大扶持力度,推进合资合作,促进60家重点企业加快发展,形成骨干财源。力争全年60家重点企业完成工业增加值85亿元,增长25%;实现主营业务收入300亿元,增长30%;纳税18亿元,增长33%。

　　推进企业自主创新。支持企业按规定足额提取研发经费,加大研发资金投入,提高企业核心竞争力。鼓励100家企业搞好产学研联合,采取自建、与高校或科研单位共建等方式,加强企业技术中心建设,新增省级技术开发中心2家以上。加快科技项目产业化,重点抓好10个自主创新项目、10个高新技术产品和10项重大科技成果转化,大力培育30家重点高新技术企业。高新技术产业产值达到140亿元,增长40%,占规模以上工业总产值的比重达到16.5%。加大企业技术改造力度,力争全年完成技改投入240亿元,增长20%。

　　继续加强银企合作。引导企业增强诚信意识,提高信用等级。严厉打击恶意逃废银行债务行为。依法完善土地使用手续,增强企业抵押贷款能力。鼓励金融部门争取上级支持,创新金融产品和服务方式。继续搞好银企合作和项目对接,确保战略合作协议实现年度化、企业化和项目化。力争全年新增贷款90亿元以上。

　　(三)全面加强新农村建设。继续把解决“三农”问题作为重中之重,认真落实各项工作措施。

　　加快发展特色板块经济和农民专业合作经济组织。各县区着力培育两三个或三四个特色产业,引导其加快向城镇集中,做大做强集特色产业、中小企业、专业市场于一体的板块经济。总结推广典型经验,加大政府扶持力度,在农副产品生产、运输、销售及农资供应、技术信息服务等方面,积极发展紧密型的农民专业合作经济组织,提高农业和农民的组织化程度。力争全年新增农民专业合作经济组织320个,达到700个左右,覆盖24万农户。

　　大力推进农业产业化经营。加大农业龙头企业培育力度,力争新增市级龙头企业45家,省级以上12家。继续强化政策扶持,搞好配套服务,推动30家重点龙头企业快速膨胀。加快优质农产品基地建设,稳定粮食总产,提高粮食品质,优质小麦、玉米面积分别达到800万亩和300万亩。积极调整农业结构,重点抓好100个农业结构调整示范村。做好林牧结合文章,力争新增造林面积30万亩,森林覆盖率提高1.2个百分点,林下养殖达到40万亩,林牧业产值分别达到150亿元和85亿元。重视发展水产事业,实现渔业总产值16亿元。

　　加快农业科技进步。推进农业良种化,争取小麦等主要作物良种率达到85%以上,主要树种、畜禽良种率分别达到55%和73%。继续实施科技特派员工程,充分发挥农业科技人员作用。搞好良种良法配套,推广先进适用技术和先进农机具,新增测土配方施肥项目县3个,农业机械化综合水平提高2个百分点,节水灌溉面积达到59万亩。抓好农业科技示范,确保高效农业示范园达到18个,科技示范网点1000个,科技示范户15万户。

　　促进农村劳动力有序转移。认真实施农民培训“阳光工程”,大力开展农民职业技能和农业技术培训,年内培训农民22万人次。完成县级人力资源市场建设,畅通农民就地转移渠道,提高劳务输出组织化程度。加大政府推动力度,打造劳务品牌。力争全年转移农村劳动力18万人。

　　深入实施“四四四一”工程。扩大“四清”范围,重点搞好乡镇驻地、学校、公路沿线的环境综合整治。加大“四改”力度,新建农村公路500公里,沼气池1.2万个,改厕30万座,新增农村自来水受益人口100万人。引导农民学文化、学技术、学政策、学法律,切实提高农民素质。编制完成300个中心村建设规划,严格宅基地管理,加快“空心村”改造。搞好农业综合开发,加强农田水利基本建设,新打机井4000眼,维修3000眼,疏浚河道1200公里,增强抗旱排涝能力。严格执行党的农村政策,继续深化农村综合改革。推行黄河水细化计量工作,严格执行“一事一议”有关规定,防止农民负担反弹。

　　(四)进一步加快城镇化步伐。完善城市规划设计。市区以城市设计为重点,加快编制城西组团、城北组团、城南组团等分区规划和控制性详细规划,搞好长江路、黄河路两侧控制性城市设计以及防汛、管线综合规划,编制洙水河公园修建性详细规划。完成体育馆、城建大厦、公安大厦、交通大厦等公共建筑和城市综合开发项目的设计。高起点编制县城专业规划及控制性详细规划,争取县城详规覆盖率提高到70%。完成乡镇总体规划,力争24个中心镇的详规覆盖率达到43%以上。

　　抓好重点工程建设。市区完成赵王河AB地块工程及大剧院主体工程;完成11条主次干道和中小道路改造;开工建设垃圾处理项目并完成主体工程,加快污水管网等配套设施建设,完成中水回用工程;引黄供水工程投入使用;推进供气、供热工程建设。搞好今冬明春的城区绿化,新建、改造绿地30万平方米。完成北外环路、人民南路至菏关高速等路灯安装,进一步完善环城公园、赵王河公园亮化工程。加快防汛设施建设,提高城市防汛能力。督导各县区抓住与群众利益密切相关、能够促进经济社会发展的重点工程,加大建设力度,完善城市功能,增强城市聚集能力和辐射能力。

　　加快城市综合开发。大力推进东西部大型居住区、南华康城、泰和水岸嘉苑、曹州大观园、香格里拉嘉园、火车站综合服务区等开发项目,努力打造精品楼盘。围绕中华路、人民路、赵王河沿线及老城区开发,精心策划论证帝都大厦、高档星级酒店等大型开发项目,积极开展招商引资。办好市住宅与房地产业博览会,提高省住博会参展水平。争取市区全年新开工房地产项目22个,完成投资22亿元,竣工面积150万平方米。搞好土地经营,搭建融资平台,为城市建设提供资金保障。

　　深入开展“城市管理年”活动。坚持建管并重,大力加强规划、净化、绿化、亮化、美化、物业等6个方面的管理,全面改善人居环境,提高居民生活质量。加大违法建设治理力度,违法建设及时查处率达到95%以上,坚决遏止乱搭乱建歪风;大力开展人行道、停车场、背街巷、马路市场、中小学周边环境、户外广告、公厕等专项治理,严肃查处乱停乱放、乱摆乱占、乱贴乱画等行为,确保年内取得明显成效。创新管理模式,理顺管理体制,完善管理制度,尽快建立城市管理长效机制。优化公交车辆,降低收费标准,确保市区公共交通更加便民利民。

　　加快交通、水利、电力等基础设施建设。实施新兖石铁路电气化改造工程,做好京九铁路电气化改造前期工作。全线开工建设德商高速菏泽段,争取济菏高速国庆节前竣工通车,做好菏徐高速前期工作;全面完成327国道改造,改造巨鄄路、单丰路等干线公路和沿黄路部分路段。搞好平原水库建设,加快推进洙水河航道改造工程。建设杜庄220千伏输变电等电力工程。争取完成基础设施建设投资33亿元。

　　(五)大力发展服务业。以50个重点服务业项目为抓手,加大招商引资力度,推动服务业快速发展。

　　加快发展生产性服务业。重点发展交通运输业,完善公路集疏运中心建设,续建、新建和改造客货运场站33个,鼓励运输企业开拓市外市场,争取全年完成货运量3720万吨。积极发展现代物流、科技服务、金融保险、信息中介、商务服务等现代服务业。做大做强商贸流通业。上半年编制完成菏泽市城市商业网点规划。加大市区牡丹路两侧专业批发市场整合力度,使花都商埠、牡丹商业步行街、康庄乐园、鲁西南建材等市场相互支撑,有机融合。加快全市9个农业市场项目建设,改造提升曹县优质板材、郓城杨木制品、鄄城中药材、成武大蒜等10大特色专业市场。全年专业批发市场交易额达到320亿元,增长20%。推广成武县农村社区综合服务中心建设经验,年内建设完成26处服务中心。继续办好农资交易会。

　　积极发展对外贸易。突出抓好单县化工、圣奥化工、佳农果蔬、曹普工艺等24家重点进出口企业,调整出口商品结构,提高出口产品档次。抓住国家鼓励进口的契机,努力扩大先进技术、重大装备和原材料进口。力争全年完成进出口7.6亿美元。

　　加快发展文化旅游业。深入开展中国优秀旅游城市创建活动,上半年编制完成菏泽市旅游总体规划。加大牡丹、水浒、武术、书画、戏曲资源开发力度,积极打造“一城三乡一生态”的旅游品牌。完善定陶仿山旅游区、鄄城孙膑旅游城基础设施,加快建设郓城水浒文化街、东明黄河森林公园,重点推进全市10个旅游项目建设。全年接待游客260万人次,实现旅游收入13亿元。

　　繁荣发展餐饮服务业。加快建设曹州名吃城,繁荣菏泽美食城。重点培育10家知名餐饮企业,加快发展连锁经营,壮大企业规模,打造餐饮特色。加强星级酒店的规范化管理,严格执法程序,提升服务水平,支持餐饮业加快发展。力争全年餐饮服务业营业额达到40亿元,增长20%。

　　(六)切实加强节能降耗和环境保护。抓好各项节能降耗指标的细化量化,逐一分解落实到各级各部门各单位,落实到项目立项、供地、供水、供电各环节。重点抓好煤炭、石油、机械、建材等行业和420家重点用能企业,推行清洁生产,发展循环经济,加快节能技术改造,淘汰一批高能耗、低效率、重污染的工艺、技术和设备。搞好企业能耗水平调查,逐一建立台账,实行在线监测,强化调度监管,确保实现节能降耗的约束性目标。加强建筑节能、交通节能和民用节能工作。大力提倡科学用能方式,增强全民节能意识。

　　逐级签订环境保护目标责任书,实行排污总量控制,执行环保“一票否决”。深入开展“整治违法排污、保障群众健康”等专项行动,严厉打击各类环境违法行为。严格执行环境影响评价和“三同时”制度,新上项目审批率、环评率和“三同时”执行率均达到95%以上。确保各县污水处理厂正常运行,县城污水集中处理率达到70%。加快推进生态市建设,深入实施“碧水蓝天”工程,加强小流域污染、大气污染和农村面源污染防治,确保三条主要河流的出境断面水质达到三类标准,城区空气质量良好率达到92%以上。及时处置环境突发事件。

　　(七)认真做好财政增收节支工作。坚持依法治税管费。以主体税种、重点行业、税源大户和新增税源为重点,加强税源管理,完善控管体系,强化社会综合治税。继续开展对餐饮、住宿、娱乐、房地产等服务行业的税收专项检查,挖掘增收潜力,确保应收尽收。全年税收收入占地方财政收入的比重提高到74%以上。加强非税收入征管,规范收费行为,将所有行政性收费、政府性基金、罚没收入等全部纳入“收支两条线”管理。搞好行政事业单位国有资产经营,加强企业国有资产监督管理,确保国有资产保值增值。

　　着力优化支出结构。按照国家统一部署,实施公务员工资制度和事业单位工作人员收入分配制度改革,提高公务员、事业单位工作人员和离退休人员的收入水平,提高伤残军人、农村五保户等各类人员的补贴标准。在保工资、保运转、保稳定的前提下,财政支出尽可能地向就业社会保障、社会事业和新农村建设方面倾斜。强化预算约束,严格支出管理,努力压缩会议、招待、交通、通讯等一般性支出;除事关全局的重大事项或突发应急事件外,一般不追加新的开支。

　　加强政府债务管理。严格实行政府举债下管一级制度,坚决限制部门举债和融资担保行为。继续抓好政府债权债务清理整顿,防范化解财政风险。

　　(八)积极构建社会主义和谐社会。抓好就业再就业和社会保障工作。认真落实就业再就业扶持政策,力争全年新增城镇就业人口9万人。开辟多种渠道,为企业下岗职工提供更多的就业机会。完善劳动合同制度,推进和谐劳动关系建设。进一步加大社会保险扩面征缴力度,完成9.7亿元的基本养老、基本医疗保险费征缴任务,努力做好失业保险金征缴工作,实现“两个确保”。关心弱势群体生产生活,提高城镇低保水平,全面启动农村低保,发展农村敬老、慈善和社会福利事业,大力实施社会救助工程。

　　优先发展教育事业。积极推进农村义务教育经费保障机制改革,从今年开始全部免除农村中小学生杂费,进一步完善“以县为主”的农村中小学教师工资保障机制。投资1.5亿元,消除农村中小学D级危房42万平方米;投资建设市区3所中小学。加快发展普通中等教育和职业技术教育,争取初中毕业生升学率达到70%以上,中职教育招生达到3万人以上,高考升学率比上年有明显提升。扩大菏泽学院招生规模,加快西校区置换和二期工程建设;支持菏泽医专搞好新校区建设;完成职业技术学院一期工程。制定优惠政策,稳定教师队伍,健全补充机制,优化师资结构,加强校风建设,进一步提高教育教学质量。

　　加快发展卫生事业。坚持医疗卫生的公益性质,加大公共卫生投入,改造乡镇卫生院108处;搞好农村卫生室和社区卫生站建设。全面铺开新型农村合作医疗,做到县不漏乡,乡不漏村,全市覆盖,尽快解决群众看病难、看病贵的问题。全面落实国家计划免疫政策,力争年内适龄儿童计划接种率达到90%以上。鼓励中医药事业健康发展。

　　积极发展文化体育等社会事业。推进30个乡镇综合文化站建设,深入开展送电影、戏曲下乡活动。继续搞好非物质文化遗产项目申报工作,启动鲁西南民俗村建设,争取《山东汉子》进入国家十大舞台艺术精品剧目。切实加强网吧、音像、娱乐等文化市场管理,严厉打击“黄赌毒”等丑恶现象。广泛开展全民健身活动,年内完成100处农村全民健身工程,启动建设承办第11届全运会散手项目的体育馆。加大竞技体育培训力度,搞好11项全省锦标赛备战参赛工作。加快推进农村有线电视建设,争取年内通有线电视的行政村达到2000个以上。认真落实计划生育基本国策,稳定低生育水平,统筹解决人口问题。加强国防教育、人民防空和民兵预备役建设,做好转业军人、退役士兵安置和双拥工作,增进军政军民团结。深入实施人才强市战略,继续引进高层次专业人才。关心支持老龄、妇女、儿童和残疾人事业,继续做好新闻出版、民族宗教、档案管理、外事侨务、社会科学、人防、史志、地震、气象等工作。

　　抓好社会稳定和安全生产。强化社会治安综合治理,加强农村、城区、单位群防群治工作,深入开展严打整治斗争,严厉打击暴力犯罪、“两抢一盗”犯罪和带有黑社会性质的有组织犯罪,进一步降低刑事发案率,努力增强广大群众的安全感。严格落实安全生产责任制,深入开展消防、道路交通、建筑施工、烟花爆竹、危险化学品等专项整治活动,确保全年安全生产事故起数、死亡人数、受伤人数和经济损失全面下降,力争杜绝重特大安全事故发生。加强基层基础工作,年内完成所有乡镇的安全生产规范化建设,危险化学品生产企业标准化建设达到80%以上。

　　认真解决损害群众利益的突出问题。大力开展专项治理活动,及时排查调处各类矛盾和纠纷,力争把问题解决在基层和萌芽状态。下更大的决心,花更大的气力,切实解决好土地征用、企业改制、房屋拆迁、环境污染、教育收费、拖欠工程款、食品药品、医药购销和医疗服务中群众反映强烈的热点难点问题,努力实现好、维护好、发展好最广大群众的根本利益。

　　三、进一步加强政府自身建设

　　按照“为民、务实、清廉”的要求,加快推进责任政府、诚信政府、阳光政府和法治政府建设,努力提高各级政府落实科学发展观、驾驭经济社会发展的能力和水平。

　　(一)推进责任政府建设。把政府抓经济工作的主要精力,放在为市场主体服务和优化发展环境上,强化服务意识,创新工作机制,认真帮助企业解决项目立项、建设用地、抵押贷款等实际问题。继续深化行政审批制度改革,清理规范审批许可项目,全面推行涉企税费统一收缴制度。加强行政服务中心建设,确保审批项目统一集中办理。加强市场监管,制止不正当竞争,严厉打击制售假冒伪劣商品行为,尤其是制售假种子、假农药、假化肥等坑农害农案件,维护市场公正和人民群众的合法权益。加强市长公开电话建设,健全三级办理网络,与信访、纠风、网络和新闻媒体配套联动,使之真正成为政府与群众的连心桥。健全突发事件应急处理机制,集中更多的物力财力向社会管理和公共服务倾斜,努力为群众提供更多更好的公共产品和公共服务。

　　(二)推进诚信政府建设。决策之前,认真论证;一旦决定,言出必践。对全年任务目标逐项细化分解,责任到人,形成一级抓一级、层层抓落实的目标责任体系。突出重点领域和关键环节,集中精力狠抓60家重点企业、10个工业园区、50个服务业项目、“四四四一”工程和重点城建项目建设,确保一抓到底,抓出成效。继续推进争创一流活动,争取全年有更多的工作走在全省前列。牢固树立正确的政绩观,健全县域经济实绩考核体系,完善考核办法,严肃查处各类弄虚作假行为,严格兑现奖惩,在全市上下营造干事创业的良好氛围。

　　(三)推进阳光政府建设。进一步深化政务公开。凡是能够公开的,都要通过新闻媒体等方式予以公开,切实维护广大群众的知情权、参与权、监督权。完善社会公示和听证制度,健全群众参与、专家咨询和政府决策相结合的决策机制,使各项决策切合实际,符合群众利益。最大限度地将政府决策的事项、过程及其结果向社会公开,便于群众了解、支持和监督。继续加强政府公报、政府网站和政策法规咨询中心建设,推动执法执纪和公益服务部门完善办事公开制度,努力为企业和群众办事创造便利条件。

　　(四)推进法治政府建设。全面实施依法行政“四五”规划,深入开展“五五”普法活动,严格按照法定权限和程序行使权力、履行职责。充分发挥纠风、行政效能投诉机构的职能作用,健全执法责任体系,加强执法监督,规范执法行为,严肃查处各类违法乱纪行为,努力提高依法行政水平。自觉接受人大的法律监督和政协的民主监督,高度重视新闻舆论监督,支持监察、审计、物价、法制等部门加强内部监督。坚持警钟长鸣,认真落实党风廉政建设责任制,严格执行中央、省、市委关于廉洁自律的各项规定。加大从源头上治理腐败的工作力度,抓住工程招投标、土地出让、政府采购、医药购销和产权交易等关键领域,着力构建完善、有效的廉政监督机制,严肃惩处腐败分子。反对红白喜事大操大办,反对公务接待铺张浪费,反对年终突击花钱,营造勤俭节约的良好社会风气。

　　各位代表!我们的政府是人民政府,所有政府公务人员都是人民的公仆。为人民服务是我们的根本宗旨,让人民满意是我们的根本追求。在新的一年里,我们一定牢记人民的期望与重托,发扬成绩,改正缺点,弥补不足,更加勤奋地学习,更加勤勉地工作;一定进一步关注民生,体察民情,爱惜民力,体恤民苦,为人民群众排忧解难,让人民群众放心满意。

　　各位代表!当前全市政治安定,社会稳定,经济发展,文化繁荣。面对这一难得的发展机遇,让我们高举邓小平理论和“三个代表”重要思想伟大旗帜,以科学发展观统领全局,在市委的正确领导下,万众一心,奋力拼搏,夺取2007年各项工作的全面胜利!
